# Pigmentation and Flavonoid Metabolite Diversity in Immature ‘Fuji’ Apple Fruits in Response to Lights and Methyl Jasmonate

**DOI:** 10.3390/ijms23031722

**Published:** 2022-02-02

**Authors:** Jung-A Ryu, Shucheng Duan, Ho-Young Jeong, Chanhui Lee, In-Kyu Kang, Seok Hyun Eom

**Affiliations:** 1 Gyeongsangbuk-do Agricultural Research and Extension Services, Daegu 41404, Korea; jaryu@korea.kr; 2 Department of Horticulture, Kyungpook National University, Daegu 41566, Korea; 3 Department of Horticultural Biotechnology, College of Life Sciences, Kyung Hee University, Yongin 17104, Korea; dsc97@khu.ac.kr; 4 Department of Plant & Environmental New Resources, College of Life Sciences, Kyung Hee University, Yongin 17104, Korea; ratank@khu.ac.kr (H.-Y.J.); chan521@khu.ac.kr (C.L.)

**Keywords:** anthocyanin, apple skin, artificial light, idaein, quercetin glycosides, ultraviolet B

## Abstract

Artificial pigmentation of apple fruits has been intensely evaluated to generate less pigmented red apples, which are profitable because of the changes in fruit quality. In this study, we analyzed the diversity of flavonoids and the patterns of flavonoid metabolic gene expression under light irradiation with or without methyl jasmonate (MeJA) treatment in immature (S1) and color-turning (S2) staged ‘Fuji’ apples. Further, we assessed the metabolic regulation at the gene level between anthocyanin and flavonol in light-responsive apple skins. UV-B exposure within 3 days was found to significantly stimulate anthocyanin accumulation in apple skin compared to other light exposure. S1 skin was more sensitive to UV-B and MeJA treatment, in the aspect of indaein accumulation. The enhancement of apple pigmentation following treatment with adequate levels of UV-B and MeJA was maximized at approximately 72 h. Red (range from 4.25 to 17.96 µg·g^−1^ DW), blue (range from 4.59 to 9.17 µg·g^−1^ DW) and UV-A (range from 3.98 to 19.12 µg·g^−1^ DW) lights contributed to the induction of idaein content. Most genes related to the flavonoid pathways increased their expression under UV-B exposure, including the gene expression of the transcription factor, MdMYB10, a well-known upstream factor of flavonoid biosynthesis in apples. The boosted upregulation of MdMYB10, MdCHS, MdF3H MdLDOX, and MdUFGT genes due to MeJA in UV-B was found and may contribute the increase of idaein. UV-A and UV-B caused higher quercetin glycoside content in both S1 and S2 apple skins than longer wavelengths, resulting in significant increases in quercetin-3-O-galactoside and quercetin-3-O-glucoside. These results suggest that the application of adequate UV-B with MeJA in less-pigmented postharvest apples will improve apple color quality within a short period.

## 1. Introduction

Epidermal color distribution in red apples is regarded as an important factor for evaluating apple quality. An appropriate skin color contributes not only the preference of consumers, but also the merit of high anthocyanin use due to various health benefit activities. The red color patterns of apple differ according to the cultivars and the presence or absence of stripes. Generally, consumers tended to select either bright red or deep red apples that lack areas with green skin, which are thought to be immature. Red apples mainly accumulate cyanidin-3-O-galactoside (idaein), a representative anthocyanin in apple skin for pigmentation, in their epidermal cells during the ripening process. Pigmentation is well known to be directly affected by environmental factors, such as altitude, temperature, and especially exposure to light [1,2]. It has been reported that expression of anthocyanin biosynthesis regulatory genes can be induced by light in red apple [3]. Both wavelength, intensity, and daily exposure time contributed to the coloration of apples. Among different wavelengths of single light sources, ultraviolet rays are known to be essential for anthocyanin accumulation in apples [4].

Improving pigmentation in apple involves the use of cultivation technology to achieve efficient light exposure, such as reflected-light setting, pruning, and chemical spraying. However, the installation of reflected-light films is often limited due to the labor and cost required to generate a wide range of orchards, and is sensitively affected by the weather conditions of the period. Chemical treatments, such as abscisic acid and ethephon, either promote deciduous leaves to improve sunlight penetration or trigger fruit ripening. Studies have recently been conducted on the anthocyanin-enhancing effect of jasmonic acid, a stress-reactive substance [5,6]. Sun et al. [7] suggested that MeJA promoted the anthocyanin accumulation in callus from red-fleshed apples, whereas abscisic acid inhibited. Rudell and Mattheis [8] reported the synergetic effect of MeJA and ultraviolet rays to enhance apple pigmentation. MeJA regulates the anthocyanin biosynthesis pathway [9]. When it was treated with MeJA (1.12 g·L^−1^) before 40 h in a UV-white light environment, the idaein content of ‘Fuji’ apple peel increased about 3 folds compared to the non-treatment control. Otherwise, the content was inhibited by either ethephon (400 µL·L^−1^) or 1-MCP (10 µL·L^−1^) treatment [10]. However, in the same experiment, a mixture of MeJA (1.12 g·L^−1^) and ethephon (400 µL·L^−1^) treatment before 40 h in a UV-white light environment, the idaein content of ‘Fuji’ apple peel increased about 6 folds. 

Anthocyanins are synthesized in the leaves and fruits of many plants, and regulated by many factors. In the biosynthetic component of anthocyanin metabolism, MYB transcriptional factors are typically known as the primary regulators of flavonoid biosynthesis [11]. Among these genes, MdMYB10 is a major transcriptional regulator that induces apple pigmentation [12]. The active gene expression of the regulatory factors enhances flavonoids, including anthocyanins, flavonones, flavanons, and flavonols. Thus, it is difficult to imply that transcription factors specifically control anthocyanins. To confirm the mechanisms of stress factors that enhance apple anthocyanin accumulation, metabolic gene expression in the flavonoid biosynthetic pathway should be assessed. In addition to gene expression, quantitative measurements of both anthocyanins and other flavonoids are essential for understanding the stress factors. Metabolic genes involved in anthocyanin synthesis from chalcones are well known in apples, including Chalcone synthase (CHS), chalcone reductase (CHR), chalcone isomerase (CHI), flavonone 3β-hydroxylase (F3H), dihydroflavonol 4-reductase (DFR), leucoanthocyanidin dioxygenase (LDOX), and uridine diphosphate-glucose: flavonoid 3-O-glycosyl- transferase (UFGT) [13]. The expression of genes in the anthocyanin biosynthesis pathway of apples, namely MdCHS, MdCHR, MdCHI, MdF3H, MdDFR, MdLDOX, and MdUFGT, is closely related to UV-B irradiation [14,15]. However, the regulation of gene expression related to pigmentation by light sources and intensities has not been evaluated in apples.

Although light and MeJA have been reported to promote pigmentation during the fruit coloring period [8,16,17], metabolic studies on the expression of anthocyanin biosynthesis genes in the flavonoid pathways and in response to light qualities and colorant chemicals are not well established. Further, quantitative studies of anthocyanin and flavonol (mainly quercetin glycosides) that accumulate via the same chalcone pathway have not been conducted. Therefore, this study was conducted to evaluate the principles of pigmentation enhancement by addressing the following: color changes of apple that are affected by single light qualities, including UV rays; specific gene expression patterns in flavonoid pathways that are induced by pigmentation enhancing light and MeJA; and apple fruit stages that are affected by pigmentation-inducing factors.

## 2. Results and Discussion

### 2.1. Determining the Light Quality for Pigmentation and Changes in SSC, pH, and Chl Content

**Figure 1:**
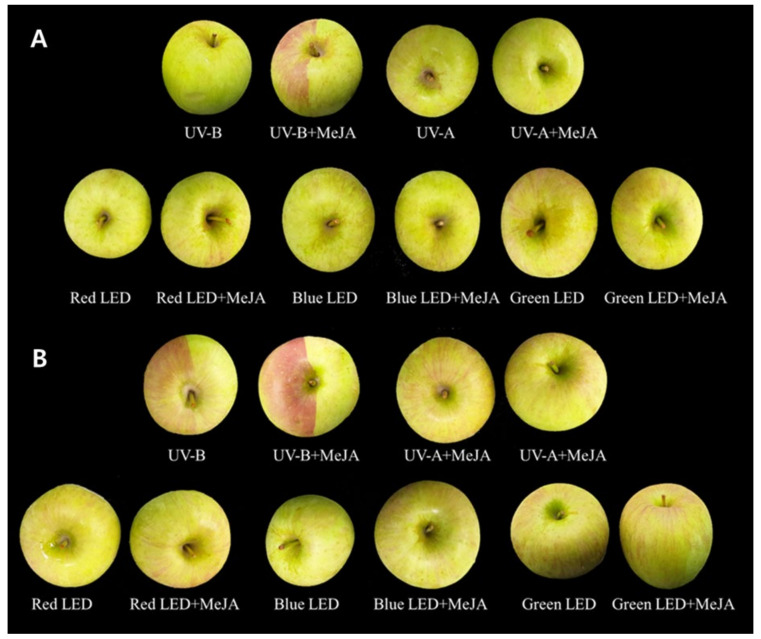
Visual symptoms of ‘Fuji’ apple skins at S1 (**A**) and S2 (**B**) affected by light sources and MeJA treatment. Half of apple skin (right side on an apple) in individual apples was initially sealed to block light penetration during 3 d light irradiation period.

Figure 1 shows the morphological changes of apple skins in S1 and S2 under different light sources, including red, green, and blue LEDs, UV-A, and UV-B with and without MeJA treatment. Single treatment with MeJA did not affect apple pigmentation, regardless of the apple stage (Figure 1). In apple fruits of S1, red-colored pigmentation was only observed following UV-B irradiation with MeJA treatment. In contrast, treatment with other lights without MeJA did not affect apple pigmentation (Figure 1). Pigmentation was observed in the epidermis of apples exposed directly to UV-B. However, a color change was not observed following UV-B irradiation without MeJA. In apple fruits of S2, visible color changes were observed following UV-B irradiation with non-MeJA and MeJA. The degree of color change was better under UV-B conditions with MeJA than without MeJA (Figure 1). Pigmentation was not observed using other lights without consideration of MeJA treatment.

Soluble solid contents (SSCs) after 3 days of light treatment were approximately 10 °Brix, with no distinct changes found among lights (Table 1). Further, apples treated with light did not distinctly differ from non-light treatment apples. The non-MeJA and MeJA treatments did not induce distinct changes in SSC. SSC is one of the main internal quality factors that determine fruit maturity and harvest period [18]. Two stages may have a similar period in SSC changes as SSC did not significantly differ between S1 (125 DAFB) and S2 (145 DAFB) ‘Fuji’ apples in this experiment.

After light treatment, the pH of apple ranged from 3.19 to 3.29 and displayed a similar pattern with the changes in SSC content (Table 1). In general, the titratable acidity of apples during maturing stages between 110 and 160 DAFB gradually decreased from 0.4% to 0.3%, which indicates an increase in pH during maturation, with no remarkable changes [19]. Thus, the pH values in the S1 and S2 apples were similar and did not change significantly in 4 days of light treatment. 

Chlorophyll content in the different apple stages was higher in S1 than S2, but did not display distinct changes among the experimental treatments. However, blue, green, and red light treatments in S2 led to higher chlorophyll content (approximately 71–138 µg·g^−1^ DW) than that induced by other light treatments (57–78 µg·g^−1^ DW) without consideration of MeJA treatment (Table 1). Although both stages had immature fruits, S1 apples tended to have higher chlorophyll content than S2 apples. According to Reay et al. [16], the chlorophyll content of ‘Gala’ apples continued to decrease between 138 and 153 DAFB. Color-changing fruits tend to have a decrease in chlorophyll content during the fruit ripening stages [20,21]. Although the color of apples changed to red following UV-B and MeJA treatments, a non-significant decrease in chlorophyll was observed during treatment. Such finding may be due to the following: chlorophyll in immature apple skins can be maintained in the relatively short UV-B exposure period, whereas chlorophyll in mature apple skins can be readily degraded by the physiochemical circumstances of the ripening process. However, anthocyanins, which are known to accumulate in the vacuoles of plant cells, are stored in apple skin, which differs from the chlorophyll accumulation site. Thus, artificially pigmented apples contain chlorophylls and anthocyanins. 

### 2.2. Accumulation of Idaein in Two Apple Maturity Stages Owing to Light Sources

**Figure 2:**
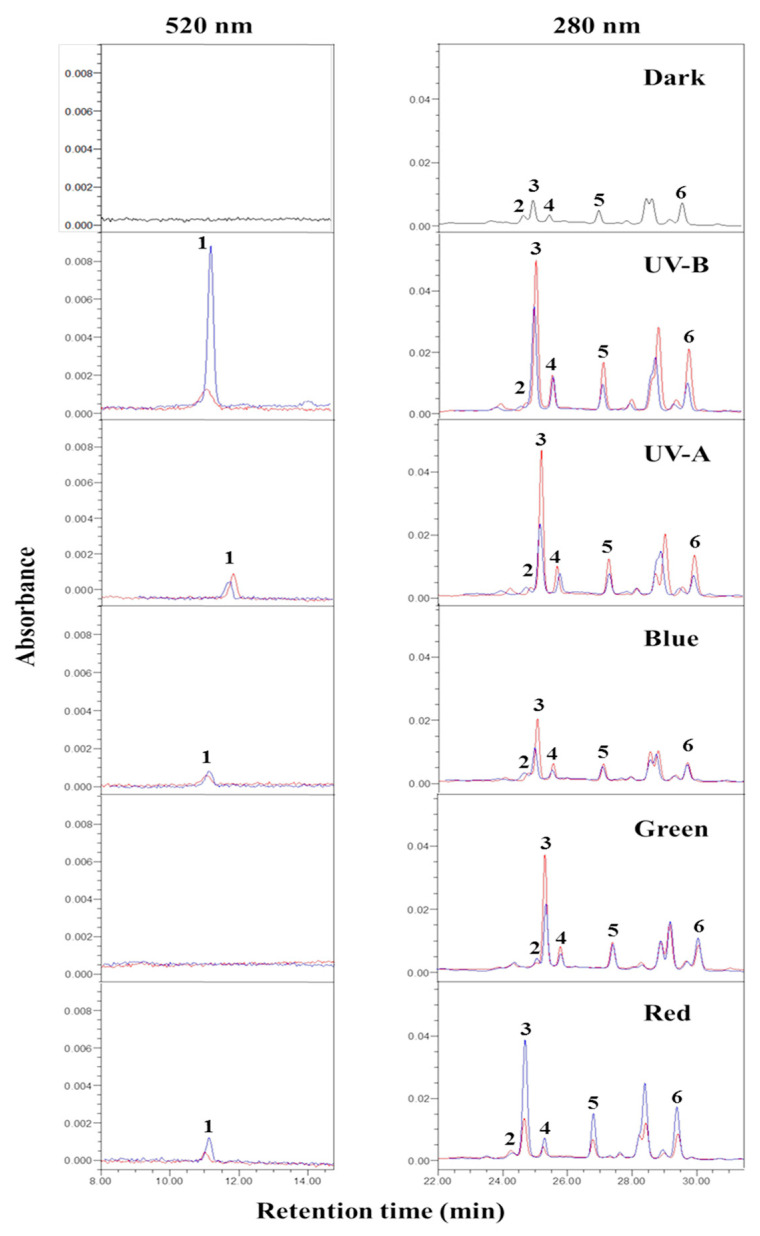
HPLC chromatograms for anthocyanin (520 nm) and flavonoids (280 nm) in the control (non-UV-B) and UV-B-exposed skins of ‘Fuji’ apples at S2. Red lines on the chromatograms indicate non-MeJA treatment control and blue lines indicate MeJA treatment samples. Each number on the chromatograms indicate idaein (1), quercetin-3-O-rutinoside (2), quercetin-3-O-galactoside (3), quercetin-3-O-glucoside (4), quercetin-3-O-xyloside (5), and quercetin-3-O-rhamnoside (6), respectively.

**Figure 3:**
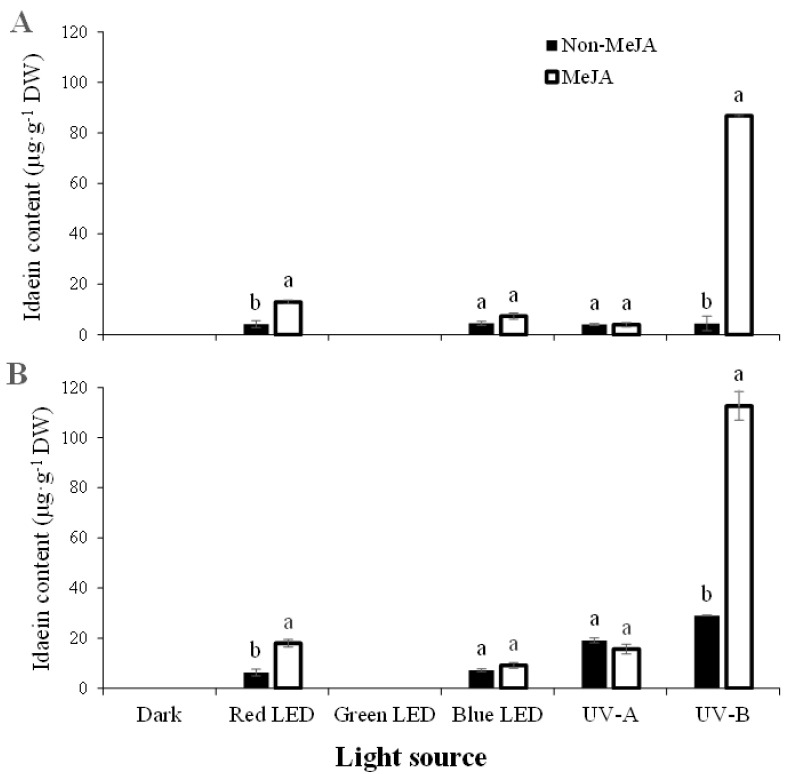
Idaein content in S1 (**A**) and S2 (**B**) staged apple skins after light irradiation treated with/without MeJA. Error bars indicate standard deviations in triplicates per treatment sample (*n* = 3). Different alphabets on bar graphs within a light are significantly differed at *p* < 0.05 on Fisher’s LSD test.

Figure 2 and Figure 3 show the light-responsive idaein content and the accumulation of idaein in two stages of ‘Fuji’ apples irradiated with different light sources for 3 days. Idaein contents in the S1 and S2 stages of apple skins under dark conditions were not detected, regardless of MeJA treatment. In S1 without MeJA treatment, idaein content did not distinctly differ among the light sources, except for green LED light, which did not affect the accumulation of idaein. In MeJA-treated S1, the content was significantly and predominantly high following UV-B irradiation (86.74 µg·g^−1^ DW). Red LED irradiation with MeJA significantly increased idaein content (12.98 µg·g^−1^ DW) compared to that obtained without MeJA (4.25 µg·g^−1^ DW), despite relatively lower increases than the treatment with UV-B and MeJA (Figure 3A). Green LED, blue LED, and UV-A irradiation did not affect the accumulation of idaein, regardless of MeJA treatment. 

In S2 ‘Fuji’ apple skins, an overall increase in idaein content was found relative to that of S1, except for the content when subjected to green LED, which did not induce accumulation (Figure 3B). In S2 without MeJA, idaein content increased under all light applications compared to S1 without MeJA, except for green LED treatment, which did not affect idaein content, similar to S1 without MeJA. Idaein content owing to UV-B without MeJA increased from 4.5 µg·g^−1^ DW of S1 to 28.99 µg·g^−1^ DW in that of S2. The idaein content significantly increased from 86.74 S1 to 112.68 µg·g^−1^ DW of S2 under UV-B irradiation with MeJA. MeJA treatment with red LED and UV-B irradiation triggered an increase in idaein content in S2, similar to S1. UV-A irradiation, regardless of MeJA treatment, resulted in increases in idaein content in S2; however, no significant difference was found between non-MeJA (19.12 µg·g^−1^ DW) and MeJA (15.64 µg·g^−1^ DW) treatments. Although the effect of MeJA was not distinct with the blue LED treatment, blue LED induced a relatively small increase in idaein content. According to Kokalj et al. [22], 7-day blue LED irradiation increased idaein content in several postharvest apple cultivars. 

**Figure 4:**
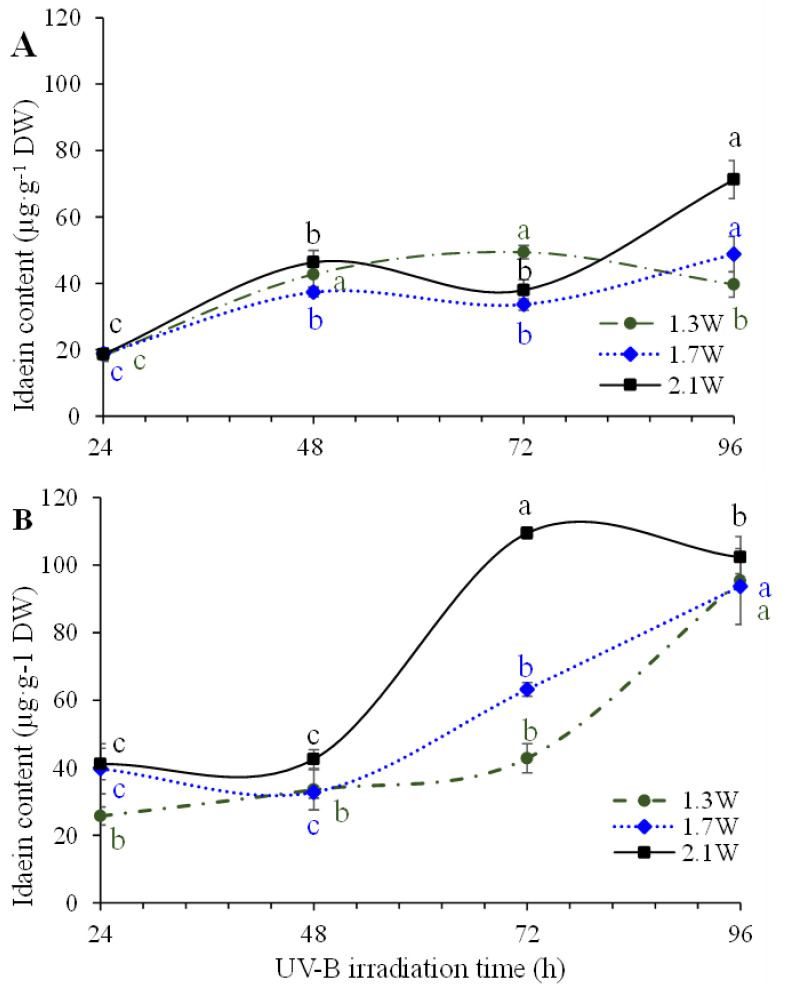
Idaein accumulation of S2 ‘Fuji’ apple skin irradiated to UV-B intensities either without (**A**) or with MeJA (**B**) treatment during the light period. Error bars indicate standard deviations in triplicates per treatment sample (*n* = 3). Different alphabets on line graphs within a UV-B intensity are significantly differed at *p* < 0.05 on Fisher’s LSD test.

As depicted in Figure 4 and Figure S1 (see Supplementary Materials), UV-B light intensities with MeJA had a strong effect on the intensity-dependent increase in idaein at 72 h, with a reduced difference in idaein content among the intensities at 96 h (Figure 4B). Otherwise, UV-B intensities without MeJA led to an intensity-dependent increase in idaein at 96 h (Figure 4A). According to the distinct effect of UV-B on anthocyanin accumulation, numerous studies have reported that UV-B irradiation is a useful source to induce anthocyanin accumulation [15,23,24]. In general, many observational studies reported that pigmentation induction owing to anthocyanin accumulation may occur quickly, ranging 1 to 4 days of light treatment [25,26,27]. Arakawa et al. [26] reported that a rapid increase in anthocyanin synthesis occurred in detached apples after 24 h of light exposure. Further, higher light intensity was reported to induce higher anthocyanin biosynthesis in apples [28]. Although the results of postharvest qualities revealed no significant damages (Table 1) following treatment with a UV-B intensity of 2.1 W·m^−2^·S^−1^, determining an appropriate light intensity and the duration of irradiation to achieve certain levels of red pigmentation is still required. Further, an appropriate UV-B intensity should be determined to avoid damages in postharvest apple qualities owing to high UV-B exposure.

Single treatment of growth regulators to apple skin, such as jasmonate and ethylene, may not be effective for the accumulation of anthocyanins in apple fruits; however, their accumulation may accelerate when it is exposed to light [17,29]. Recent studies have reported that MeJA affects de-greening of apple skins and promotes anthocyanin accumulation, suggesting a synergetic reaction with either light exposure or abscisic acid [5,6,8]. This study clearly shows that MeJA distinctly acts as an efficient inducer of apple color stimulation with UV-B synergism, leading to approximately 20-fold (S1) and 4-fold (S2) higher idaein accumulation via UV-B single treatment. In contrast, MeJA did not strongly stimulate apple coloration under other light sources (Figure 3). Therefore, 72 h of treatment with UV-B (2.1 W·m^−2^·S^−1^) and MeJA (1.12 mg·L^−1^) was concluded to be the suitable condition for achieving a high idaein content in apple skins. According to the findings of this experiment, the increase in idaein in red apples is affected by various conditions, such as apple fruit stages, UV-B intensities, colorants, and treatment duration. These conditions are closely related to each other in the coloration of red apples.

**Figure 5:**
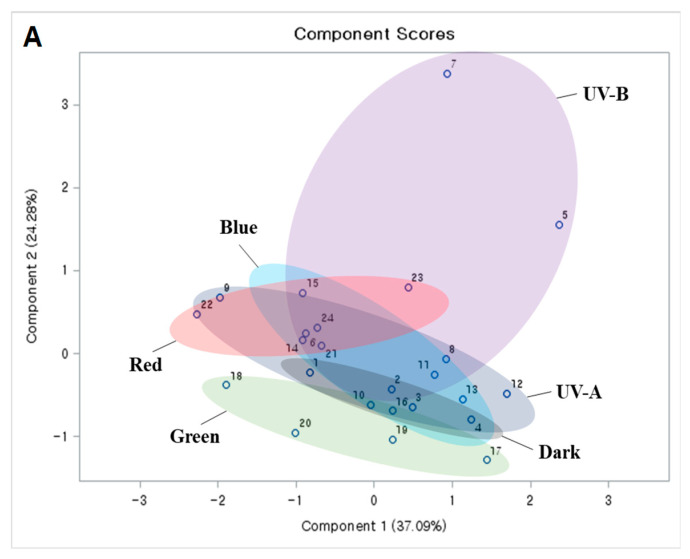

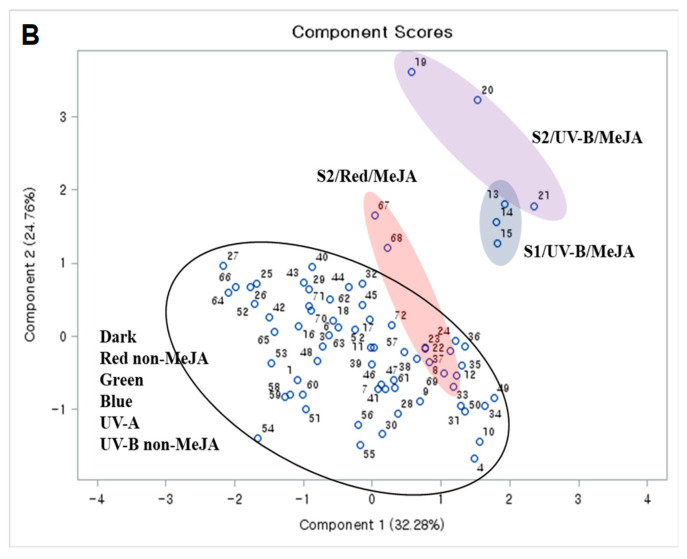
Principal component analysis score plots analyzed between light sources (**A**) or between light sources with/without MeJA (**B**) in S1 and S2 stages of apples. The score plots were generated from the data of SSC, pH, total chlorophyll content, and idaein content.

**Figure 6:**
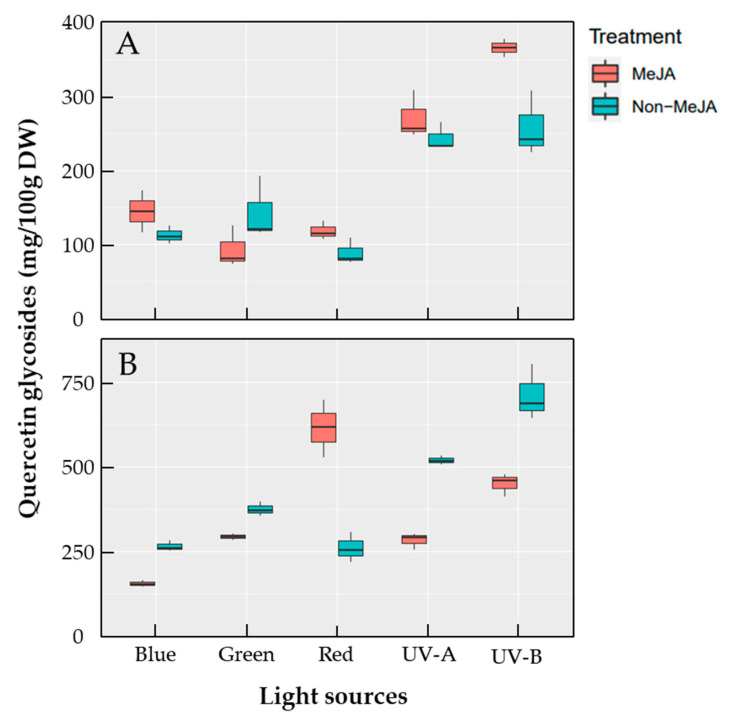
Content changes of total quercetin glycosides in S1 (**A**) and S2 (**B**) ‘Fuji’ apples after 3 d light irradiation either Non-MeJA or MeJA treatment.

To confirm the effects of light and MeJA on apple component changes, principal component analysis (PCA) was performed with two types of apple data. However, SSC, pH, and total chlorophyll content data did not significantly differ among light treatments (some differences are observed in apple stages) while idaein content data significantly differed among the light treatments and apple stages. Figure 5 shows the results of PCA generated from SSC, pH, total chlorophyll content, and idaein content in S1 and S2 apple skins in response to either light source (Figure 5A) or the effect of MeJA under light (Figure 5B). UV-B showed distinct factors to the apple data examined, while other light sources did not show clear patterns that were arranged at similar distances on PCA (Figure 6A). UV-B treatments with MeJA led to distinct scores for S1 and S2 apples compared to other light treatments with/without MeJA and UV-B treatments without MeJA. When S1 and S2 were compared under UV-B with MeJA, the PCA plot scores were found to be distinct, with differences in the effect of apple pigmentation induced by UV-B plus MeJA with respect to fruit maturity. The plot scores of S2 apples treated with red light and MeJA showed slight differences, as shown in Figure 6B. The different scores resulting from the variance of idaein content indicate that the red light source with MeJA induces apple pigmentation, despite a lower pigmentation than that of UV-B light with MeJA.

### 2.3. Expression of Genes Involved in Flavonoid Biosynthesis in UV-B-Treated Apples

Flavonoids are synthesized via the shikimate pathway and are initially produced as phenylalanine. CHS forms chalcones by combining p-coumaroyl-CoA and malonyl-CoA. Anthocyanins and various types of flavonoids are synthesized from chalcones via the expression of several metabolic enzymatic genes, such as CHR to form naringenin chalcone; CHI to form naringenin; F3H to form dihydroflavanone; DFR to form flavan-3,4-diol; LDOX to form 3-OH-anthocyanidins; and UFGT to form anthocyanins. The enzymatic gene expression related to flavonoid biosynthesis was evaluated in ‘Fuji’ apples administered UV-B with/without MeJA. The metabolic enzymes in the anthocyanin biosynthetic pathways were CHS, CHI, F3H, DFR, LDOX, and UFGT in Malus domestica (Md). 

**Figure 7:**
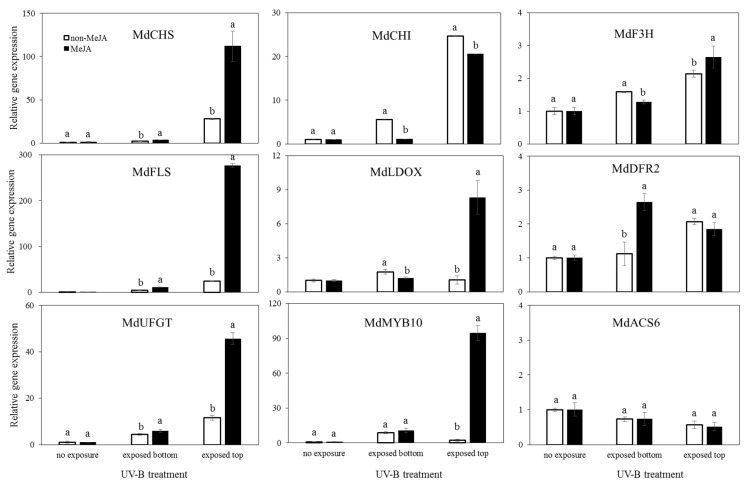
Relative gene expressions of flavonoids biosynthetic metabolisms in S2 apple skins by UV-B light irradiation effects with/without MeJA. Aberrations indicate MdCHS (chalcone synthase), MdFLS (flavonol synthase), MdLDOX (leucoanthocyanidin dioxygenase), MdUFGT (uridine diphosphate-glucose:flavonoid 3-O-glycosyltransferase), MdCHI (chalcone isomerase), MdF3H (flavanone 3β-hydroxylase), MdDFR2 (dihydroflavonol 4-reductase 2), MdMYB10 (Myeloblastosis 10), and MdACS6 (1-aminocyclopropane-1-carboxylate synthase 6), respectively. Error bars indicate standard deviations in triplicates per sample treatment (*n* = 3). Different alphabets (a,b) on bar graphs within an apple skin part are significantly differed at *p* < 0.05 on Fisher’s LSD test.

Figure 7 shows the relative gene expression among apple skins with UV-B-exposed top skin, UV-B-exposed bottom skin, and UV-B-non-exposed skin, respectively. Under non-UV-B exposure, MeJA treatment slightly increased MdCHI and MdLDOX gene expression, with a 2.2-fold increase in MdCHI and 1.7-fold increase in MdLDOX compared to non-MeJA treatment. However, the significant increases were relatively low. The expression of other genes was not increased by MeJA treatment. For UV-B exposed top skins regardless of MeJA treatment, the expression levels of all genes were significantly increased. The expression levels of all genes, except MdDFR2, were maximized by UV-B exposure with MeJA treatment. The expression of the MdDFR2 gene presented the highest value (2.1-fold of non-UV-B without MeJA) under UV-B without MeJA, and a lower value (1.3-fold) under UV-B with MeJA. The expression levels of other genes were significantly increased in the MeJA-treated UV-B exposed top skins, with 45.1-fold for MdCHS, 45.3-fold for MdCHI, 2.8-fold for MdF3H, 56.8-fold for MdFLS, 14.1-fold for MdLDOX, and 22.4-fold for MdUFGT. When non-MeJA and MeJA treatments under UV-B exposure were compared, the gene expression levels of MdCHS, MdCHI, MdFLS, MDLDOX, and MdUFGT were significantly increased by 1.6-, 1.8-, 2.3-, 13.6-, and 1.9-fold, respectively, in the MeJA-treated skins.

The indirect effects of UV-B, in terms of the UV-B-exposed bottom part of an apple, revealed a certain increase in enzymatic gene expression in most enzymes evaluated; however, the increased levels were quite low compared to the expression levels found in UV-B-exposed top skin in apples. When the UV-B-exposed bottom skins were compared, UV-B was found to induce an increase in gene expression, except for MdDFR2. For the UV-B and non-UV-B comparison under MeJA treatment, the expression of all genes was slightly increased. Interestingly, MdDFR2 expression in the exposed bottom skin was higher than that in the exposed top skin. Thus, most genes were significantly overexpressed in the UV-B exposed top apple skins, which were directly affected by UV-B irradiation, except the gene expression in UV-B without MeJA of MdLDOX and UV-B with MeJA of MdDFR2 compared to the UV-B exposed bottom skins. Numerous studies reported that UV-B irradiation and sunlight increase the expression of certain flavonoid metabolic genes [4,14,15,30]. Further, MeJA was found to upregulate MdCHS, MdF3H, and MdUFGT in the leaf callus of apples and increase the expression of the metabolic genes involved in anthocyanin synthesis [7,31]. According to Feng et al. [31], MeJA induces an idaein increase of approximately 7-fold in MeJA-treated ripening apples and triggered 4-to-20-fold upregulation of F3Hs and UFGTs compared to non-MeJA apples. Qian et al. [32] revealed that the changes in anthocyanin biosynthetic gene expression in pears were identified as whether UV-B and MeJA independently affected the changes. The study exhibited the expression patterns of UV-B and MeJA synergism, resulting in the upregulation of PpCHS1, PpDFR1, PpANS, and PpUFGT. However, in apples, the gene expression changes were not clearly defined before this study to delineate the effect of synergism between UV-B and MeJA, despite studies on the impact of the synergetic effect on anthocyanin accumulation in apples [8]. Based on the results of this study, the anthocyanin biosynthesis genes in response to UV-B with MeJA were successfully categorized, with strong upregulation of MdMYB10, MdCHS, MdLDOX, and MdUFGT identified (Figure 6). Collectively, the results clearly indicate that anthocyanin biosynthesis genes were not upregulated in apple skins treated with MeJA without UV-B irradiation.

The gene expression of MdMYB10, a transcriptional factor in a clade of R2R3 MYB transcriptional factors that are known to control anthocyanin biosynthesis in apples [33], was significantly increased in UV-B with MeJA-exposed apple skins, with 95-fold higher levels than those found in UV-B non-exposed apple skins (Figure 6). However, gene expression was not distinctly increased in UV-B irradiation without MeJA treatment. Red coloration in apples is due to the activity of the MYB transcription factor, MdMYB 10 [8]. The findings in this study suggest that MdMYB10 overexpression resulted in apple pigmentation due to the synergistic effect between UV-B and MeJA (Figure 6). MdMYB transcription factors are known to be regulated by MeJA in apple calli [34]. In addition, MeJA treatment increases the expression of metabolic genes in the anthocyanin pathway [31]. Therefore, the upregulation of MdMYB10 might result from the synergetic effect of UV-B and MeJA. 

Ethylene biosynthesis is a complicated process that involves the cooperation of several ACS genes [35,36]. During the development of ‘Golden Delicious’ apple, MdACS6 was initiated at an earlier stage (70 DAFB), whereas MdACS3a and MdACS1 began to be expressed at 35 d before harvest and immediately after harvest, respectively [37]. In this experiment, the MdACS6 gene expressions, known to one of gene activating 1-aminocyclopropane-1-carboxylate synthase that is an essential enzyme for ethylene synthesis, did not respond to either UV-B irradiation or MeJA treatment in apple skins (Figure 6). The expression was decreased in UV-B-exposed top apple skins. Although MeJA promotes ethylene biosynthesis and enhances MdACS genes during postharvest apple storage under ambient conditions [38], the synergistic effect of UV-B and MeJA might not be elucidated for ethylene synthesis in postharvest immature apples. Generally, ethylene synthesis in fruits is vigorously stimulated during the fruit ripening process; thus, immature fruits even under short (within 3 days) UV-B stress did not generate ethylene biosynthesis. A more detailed study is needed to determine the effect of UV-B and MeJA treatment on immature apples in the production of ethylene in post-harvest apples.

### 2.4. Accumulation of Quercetin Glycosides in Apple Skins Exposed to Lights

**Figure 8:**
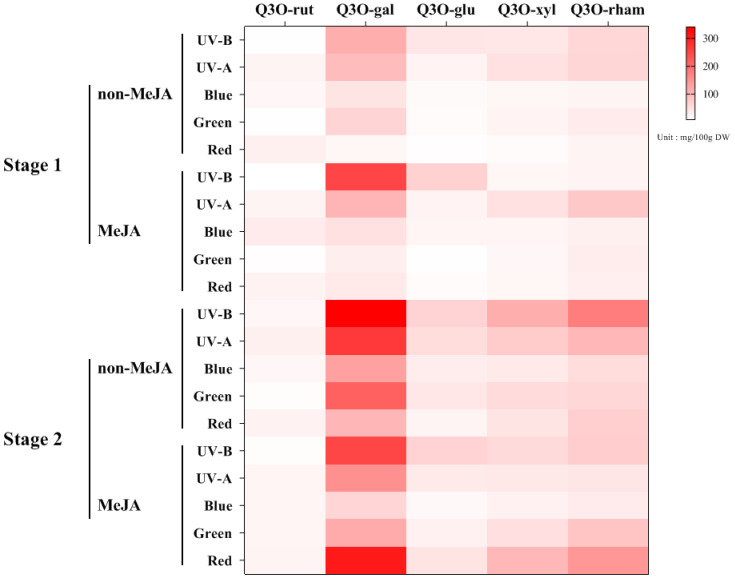
A heatmap of quantitative changes of quercetin glucosides in S1 and S2 ‘Fuji’ apple skins exposed to lights with/without MeJA for 3 d. Q3O-rut, Q3O-gal, Q3O-glu, Q3O-xyl, and Q3O-rham indicate quercetin-3-O-rutinoside, quercetin-3-O-galactoside, quercetin-3-O-glucoside, quercetin-3-O-xyloside, and quercetin-3-O-rhamnoside, respectively.

Five quercetin glycosides were detected in ‘Fuji’ apple skins by HPLC analysis (Figure 2). Quercetin glycosides tended to show overall increases when apple maturation progressed from S1 to S2. The highest content among the quercetin glycosides was quercetin-3-O-galactoside, from 19.34 to 250.37 mg·100 g^−1^ DW in S1 and 62.07 to 341.15 mg·100 g^−1^ DW in S2 under different light exposures (Table 2 and Table 3). The high increase in quercetin-3-O-galactoside, from 34.45 to 250.37 mg·100 g^−1^ DW, was observed in the S1 apple skins subjected to UV-B with MeJA treatment (Table 2). In S2 apple skins, a high increase in quercetin-3-O-galactoside, from 44.39 to 341.16 mg·100 g^−1^ DW, was observed in UV-B without MeJA (Table 3). Therefore, a relatively high accumulation of quercetin-3-O-galactoside was observed with UV-B irradiation, regardless of MeJA treatment. UV-B without MeJA, UV-A without MeJA, UV-B with MeJA, and red light with MeJA treatments in S2 apple skin affected the high accumulation of quercetin-3-O-galactoside (range > 248 mg·100 g^−1^ DW) (Figure 8). Interestingly, five quercetin glycosides of red light-exposed apple skins (S2) were highly accumulated owing to MeJA treatment. However, compounds of other light-treated skins showed relatively higher accumulation in non-MeJA treatments (Figure 8). The quantitative amounts of other quercetin glycosides varied according to light exposure, with quercetin-3-O-rutinoside, 7.63–32.56 mg·100 g^−1^ DW (S1) and 10.34–25.99 mg (S2); quercetin-3-O-glucoside, 8.02–67.03 mg (S1) and 16.06–64.89 mg (S2); quercetin-3-O-xyloside, 12.02–46.50 mg (S1) and 24.79–112.92 mg (S2); and quercetin-3-O-rhamnoside, 20.84–78.46 mg (S1) and 32.62–176.42 mg (S2) (Table 2 and Table 3). 

Light sources affect the accumulation of total quercetin glycosides in different manners. In S1, light-exposed skins had high accumulation of total quercetin glycosides compared to non-light-exposed skins, except for green and red lights without MeJA treatment and blue light with MeJA treatment (Table 2). With respect to individual light sources related to the contents of quercetin glycosides, UV-B and UV-A irradiation, regardless of MeJA treatment, induced relatively higher contents of the compounds. These results agree with the notion that shortened light wavelengths, such as ultraviolet rays, in the range of 320–650 nm, more efficiently trigger flavonol accumulation. According to previous studies, the effects of UV-B and other lights increase the levels of quercetin glycosides [23,39,40]. The total quercetin glycoside content in S2 was higher than that in S1. When the contents owing to MeJA and non-MeJA treatments were compared, non-MeJA treatments with light irradiation produced more quercetin glycoside contents, except for non-MeJA with red light irradiation (615.09 mg·100 g^−1^ DW) and MeJA with red light irradiation (260.81 mg) (Table 3). The increase in quercetin glycosides is controlled by the degree of red light irradiation and is critically determined by the ratio of far-red and red rays [41]. With blue light treatment, quercetin glycosides accumulated in a different manner between apple of different stages. In stage 1, blue light treatment significantly increased the levels of four quercetin glycosides but significantly decreased that of quercetin-3-O-rutinoside (Table 3). In stage 2, light treatment significantly increased quercetin-O-galactoside and quercein-3-O-glucosides, while treatment decreased the levels of other glycosides (Table 3). These results indicate that the accumulation patterns of quercetin glycosides are dependent on the fruit stage. Using blue light irradiation, Kokalj et al. [22] revealed similar results to S2 in this study.

Figure 8 shows the changes in the content of total quercetin glycosides in the two stages of apple skin irradiated with light sources with/without MeJA. Light irradiation without MeJA increased the content of total quercetin glycosides. Further, shorter rays were found to produce more content. In contrast, UV-rays with MeJA showed significantly higher total quercetin glycosides content than other lights in S1 (Figure 8). All lights, except green light, produced high total quercetin glycosides with MeJA compared to non-MeJA (Figure 8). Light wavelengths in S2 also affected the accumulation of quercetin glycoside content in light without MeJA (Figure 8). The graphs clearly show that UV-B irradiation produces more quercetin glycosides and exhibits a synergetic effect with MeJA in S1 apple skins, but induced less quercetin glycoside accumulation with MeJA in S2 apple skins. Red light with MeJA resulted in an exceptionally high accumulation of quercetin glycoside content compared to other light sources with MeJA, which may be explained by the red light effect described by Awad et al. [41]. Therefore, shorter wavelengths may induce higher accumulation of quercetin glycosides in apple skins, except red light treatment with MeJA.

## 3. Materials and Methods

### 3.1. Apple Harvest

‘Fuji’ apples, about 20 year-old trees placed in an orchard (N 37°14′36.0″, E 127°04′52.6″), Yongin, Korea, were harvested at early (Stage I, S1) and late September (Stage II, S2) in 2020. The apple stages were about 125 and 145 days after full blooming (DAFB), respectively, which present green skin in S1 and slightly reddish-green skin in S2 apples.

### 3.2. Light and MeJA Treatments

The individual apples harvested were vertically sealed about 50% with a commercial aluminum foil. Nine apples in a treatment were placed into a light transparent plastic bag (32 × 33 cm, Ziploc^®^, Mexico Jonhson, Toluca, Mexico). Each 20 mL of solution prepared to 1.12 mg·L^−1^ of MeJA (Sigma-Aldrich, St. Louis, MO, USA) with 3 drops of tween 20 was sprayed into each plastic bag and sealed. The applied lights were five individual light sources, including red LED light (λmax = 620 nm, P5II models supplying 3.3 V and 1 W per module; Seoul Semiconductor, Seoul, Korea), green LED light (λmax = 520 nm), blue LED light (λmax = 460 nm), UV-A light (λmax = 370 nm, F71T12 100W, Philips Co., Hamburg, Germany), UV-B light (λmax = 320 nm, Narrowband TL 20W/01—RS Ulterviolet-B, Philips Co.), respectively. The light intensities of red, and blue were set to 200 μmol·m^−2^·s^−1^ on the surface of apple skins. The light intensity of green was set at 40 μmol·m^−2^·s^−1^. The light intensities of UV-A and UV-B were kept to 2 W·m^−2^·s^−1^ (1 W·m^−2^·s^−1^ = 4.59 μmol·m^−2^·s^−1^). The light intensities of each light were controlled by measuring inside plastic bag on the light with a photo-radiometer (HD 2302.0, Delta OHM SRL, Marconi, Italy). All light treatments were performed at 23 ± 2 °C For the MeJA effect under UV-B light, the apple bags with/without MeJA treatment were placed into a UV-B chamber set to 2 W·m^−2^·s^−1^. Exposed apple parts (not-sealed part of an apple) were set toward UV-B light when the plastic bags were placed in the UV-B chamber (Figure 1). All treatments were performed in triplicate.

### 3.3. Sample Preparation and Extraction

After 3 days of light treatment, apples were harvested and quickly rinsed with distilled water and eliminated to water by a commercial paper towel. Nine apples of each treatment were randomly divided into two groups: four apples were gathered for analyzing metabolic gene expression; five apples were gathered for analyzing flavonoids. After peeling aluminum foil from apples, three parts of apple tissues, UV-B exposed top skin, UV-B less exposed bottom skin, and non-UV-B exposed skin were cut by < 2 mm thickness and placed into 50 mL tubes. For analyzing metabolic gene expression, the collected samples were immediately frozen with liquid N_2_ and stored at a −68 °C freezer before conducting RNA extraction for RT-PCR analysis. For analyzing flavonoids, the collected samples were directly stored at a −68 °C freezer. The frozen samples were lyophilized with a vacuum freeze dryer (IlshinBioBase. Co. Ltd., Dongducheon, Korea) and kept to the samples in a −15 °C freezer until conducting further analysis. Apple fleshes were ground using commercial grinder and measured using refractometer (Atago Co., Tokyo, Japan) for measurement of soluble solid content (SSC). In addition, pH of the saps was measured. Total chlorophylls in 1 mg of freeze dried apple skins were extracted using 80% acetone (1 mL) for 24 h. Absorbance of the extract was measured at 645 and 663 nm, respectively, using a spectropho-tometer (S-4100, Scinco, Seoul, Korea). The total chlorophyll content was calculated by a previously described method [42].

### 3.4. HPLC Analysis of Flavonoids

The lyophilized apple skins and fleshes were coarsely ground with a pestle and a mortar. Each 50 mg of the ground samples was dissolved in 1 mL of 80% ethanol (*v*/*v*) with 1% formic acid and extracted by ultra-sonication at 40°C for 1 h. The supernatants after 14,000 rpm centrifugation of the mixtures for 5 min were harvested and filtered through a 0.45 µm membrane syringe filter (Futecs Co., Daejeon, Korea). The extracts were analyzed using a reverse phased high performance liquid chromatography (HPLC, Waters 2695 Alliance model, Waters Inc., Milford, MA, USA). Injection volume of the samples was 5 µL. Column used was an octadecylsilane column (Prontosil 120-5-C18-SH 5.0 µm, 250 × 4.6 mm, Bischoff, Leonberg, Germany). The column temperature was kept to 30 °C. The mobile phase through HPLC system was a combination of (A) water with 0.1% trifluoroactic adid (TFA) and (B) acetonitrile with 0.1% TFA. The gradient elution was performed by 90% of solvent A at initial time, 85% at 5 min, 83% at 19 min, 79% at 21 min, 76% at 34 min, 50% at 35 min, 10% at 41, and 90% at 43-45 min, respectively. The flow rate of the mobile phase was maintained to 1.0 mL per min. Peaks were monitored at 520 nm for anthocyanin analysis and at 280 nm for quercetin derivatives using a photodiode array detector (Waters 996, Waters Inc.). The standards of cyanidin-3-O-galactoside chloride (idaein) and quercetin-3-O-glucoside were purchased from Sigma-Aldrich (St. Louis, MO, USA). The quantitative analysis of other quercetin derivatives was calculated on the basis of quercetin-3-O-glucoside standard.

### 3.5. Analysis of Metabolic Gene Expressions Related to Flavonoid Biosynthesis

Apple tissues stored at −70 °C after light and MeJA treatments were ground in liquid nitrogen using a mortar and a pestle treated with RNaseZap (Thermo Fisher, Waltham, MA, USA). The Direct-zol RNA Miniprep Plus kit (Zymo Research, Irvine, CA, USA) and Trizol reagent (Thermo Fisher) were used for total RNA extraction from the ground tissues. At the same time, DNase was treated to remove genomic DNA. The first strand of cDNA was synthesized using oligo (dT) primers with the ProtoScript M-MuLV Taq RT-PCR kit (New England Biolabs, Ipswich, MA, USA). All reactions were conducted in triplicate and the MdActin primer sequence was used as the internal control (Table S1). Cycling conditions were initial 180 sec at 95 °C; then 40 times of repeats for each 30 s at 95 °C, 30 s at 55 °C, and 60 s at 72 °C, following the melt-curve analysis. Primers used for the PCR were shown in Table 1. The cycle threshold values were calculated using CFX manager (Bio-Rad). The relative expression values via non-light exposed skins were calculated using the 2–∆∆Ct method. Quantitative PCR (qPCR) was conducted using the CFX Connect Real-Time PCR Detection System (Bio-Rad, Hercules, CA, USA) with iTaq Universal SYBR Green Supermix (Bio-Rad). Each 20 μL quantitative reverse transcription (qRT)-PCR contained 10 μL SYBR Green Supermix, 5 μL cDNA template (1 ng·μL^−1^), and 0.5 μL of each primer (10 μM). 

### 3.6. Statistical Analysis

All data of tables and figures in this chapter were shown using averages with standard deviations obtained from triplicates per each sample treatment (*n* = 3). Statistical analysis in the experimental data were performed using SAS program (Enterprise guide 4.3 version, SAS Institute Inc. Cary, NC, USA). The significant differences among averages of treatments, presenting alphabetical classify, were categorized using Fisher’s least significant difference (LSD) test at the level of probability value less than 0.05 (*p* < 0.05). A heatmap was performed with the contents of quercetin glycosides in 100 g dry weight of apple skins, which were normalized data (mg/100 g dry weight (DW)) generated by 4.0 version of MetaboAnalyst (www.metaboanalyst.ca, accessed on 20 December 2021).

## 4. Conclusions

**Figure 9:**
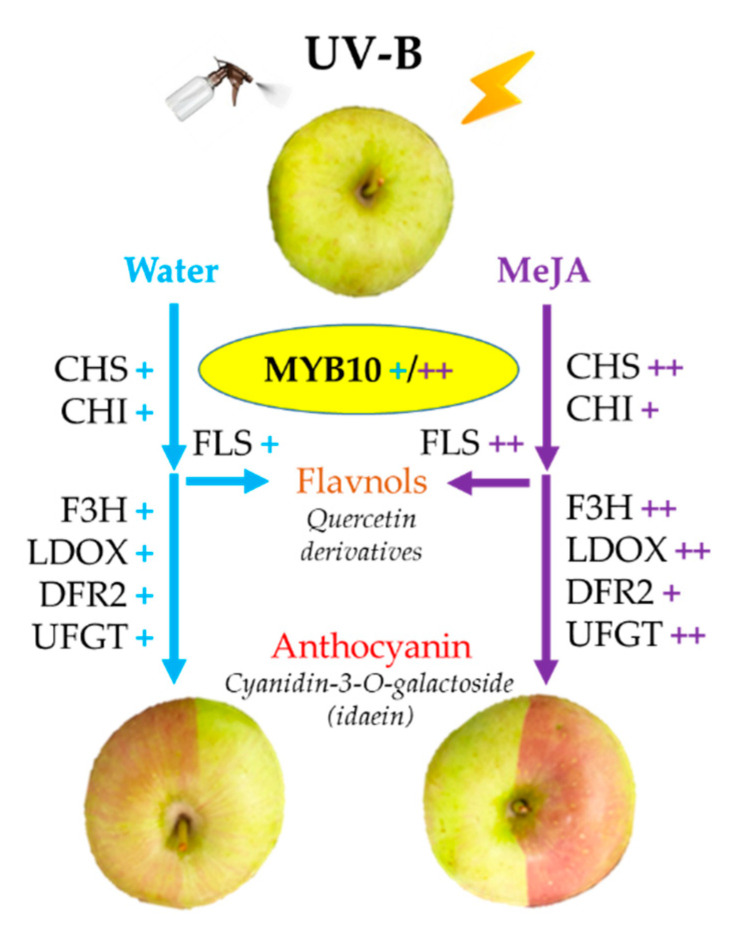
Flow scheme of flavonoid gene expression in apple coloration. + and ++ indicate overexpression by UV-B and boosted-overexpression by UV-B with MeJA, respectively.

In the aspect of overall results (Figure 9), it was confirmed that the pigmentation of epidermal skin in postharvest apples could be rapidly performed by appropriate treatment of UV-B with MeJA. The pigmentation effect of the shor-term artificial treatment presented rapidly in less mature apple skins. Most of genes related to flavonoid pathways increased their expressions under UV-B exposure. The boosted up-regulation of MdMYB10, MdCHS, MdF3H MdLDOX, and MdUFGT genes due to MeJA in UV-B was found and may contribute the increase of idaein. It was found that the boosted up-regulation of MdMYB10, MdCHS, MdF3H MdLDOX, and MdUFGT genes due to MeJA in UV-B, which may contribute the increase of idaein. Flavonoids, such as anthocyanins and quercetin glycosides were mostly increased by UV-B irradiation with MeJA. The results of this study will contribute to the successful early establishment of postharvest apple pigmentation technology by providing basic knowledge to the study to investigate the correlation between MeJA concentration and the more detailed UV-B intensity and exposure time control.

## Figures and Tables

**Table 1:** Soluble solid content (SSC) and pH changes of S1 and S2 ‘Fuji’ apples exposed to lights for 3 days.

Light	MeJA ^z^	SSC (°Brix)		pH		Total Chl (µg∙g^−1^ DW)	
		S1	S2	S1	S2	S1	S2
Dark	O	9.7 abcd ^y^	9.8 abc	3.2 b	3.2 a	89.3 bc	64.5 fg
	X	9.7 abcd	9.8 ab	3.2 ab	3.3 a	67.5 c	61.6 fg
UV-B	O	9.9 a	9.6 abcd	3.3 a	3.2 a	103.1 b	78.4 def
	X	9.5 cd	9.9 a	3.2 ab	3.2 a	87.5 bc	76.3 def
UV-A	O	9.4 d	9.8 d	3.3 ab	3.2 a	142.9 a	65.0 fg
	X	9.8 abc	9.9 a	3.2 ab	3.3 a	96.4 b	56.7 g
Blue	O	9.8 abc	9.4 d	3.3 a	3.2 a	96.5 b	85.5 cde
	X	9.6 bcd	9.9 ab	3.2 ab	3.2 a	101.5 b	109.8 b
Green	O	10.0 a	9.9 a	3.3 a	3.2 a	100.5 b	94.2 bcd
	X	9.7 abcd	9.9 a	3.2 b	3.2 a	128.3 a	138.8 a
Red	O	9.7 abcd	9.5 cd	3.2 b	3.3 a	85.2 bc	71.1 efg
	X	9.5 cd	9.5 bcd	3.2 b	3.2 a	132.0 a	97.8 bc
LSD values		0.5789	0.3463	0.0701	0.052	22.782	18.099

^z^ O and X indicate MeJA and non-MeJA treatments, respectively. ^y^ Different small alphabets next to values (averages of triplicates, *n* = 3) within a column and different capital letters within each stage of measurements are significantly differed at *p* < 0.05 on Fisher’s LSD test.

**Table 2:** Accumulation of quercetin glucosides in S1 ‘Fuji’ apple skins after light irradiation with/without MeJA. Unit: mg∙100 g^−1^ DW.

MJ	Light	Quercetin-3-O-Rutinoside	Quercetin-3-O-Galactoside	Quercetin-3-O-Glucoside	Quercetin-3-O-Xyloside	Quercetin-3-O-Rhamnoside	Total Quercetin Derivatives
non-MeJA	UV-B	8.26 ± 0.19 f	113.34 ± 12.18 a	39.45 ± 3.75 a	38.74 ± 4.21 b	59.08 ± 5.27 a	258.87 a
	NE	11.49 ± 0.28 ef	18.66 ± 4.43 fg	6.91 ± 1.73 ef	11.89 ± 1.59 ef	27.54 ± 3.23 cd	79.49 cd
	UV-A	20.95 ± 1.50 cd	94.92 ± 5.05 b	22.05 ± 1.53 b	46.50 ± 1.80 a	60.01 ± 1.13 a	244.43 a
	NE	26.27 ± 2.16 a	34.94 ± 1.56 ef	15.65 ± 2.24 c	19.23 ± 1.36 cd	37.10 ± 0.96 c	133.19 b
	Blue	17.82 ± 0.95 d	42.69 ± 3.01 de	13.16 ± 1.00 cd	18.21 ± 1.13 de	20.84 ± 0.95 d	112.72 bc
	NE	21.97 ± 0.94 bc	6.68 ± 0.57 g	3.40 ± 0.29 f	6.44 ± 0.19 f	10.55 ± 0.40 e	49.04 d
	Green	12.43 ± 1.34 e	62.68 ± 11.28 c	12.13 ± 2.15 cde	23.08 ± 4.05 cd	33.22 ± 6.00 c	133.20 b
	NE	12.12 ± 0.78 e	54.21 ± 1.60 cd	11.03 ± 0.85 cde	24.90 ± 0.38 c	48.11 ± 0.95 b	150.37 b
	Red	26.02 ± 1.52 a	19.34 ± 2.40 fg	9.61 ± 2.09 de	12.02 ± 1.29 ef	21.87 ± 3.97 d	88.86 cd
	NE	25.35 ± 1.77 ab	23.41 ± 4.32 fg	7.73 ± 0.91 def	19.10 ± 2.31 cd	36.18 ± 4.31 c	111.77 bc
LSD values		3.81	17.69	5.60	6.61	9.91	40.87
MeJA	UV-B	7.63 ± 0.01 d	250.37 ± 13.65 a	67.03 ± 0.03 a	19.38 ± 0.15 c	22.27 ± 1.21 cd	366.68 a
	NE	9.36 ± 0.76 d	34.45 ± 4.21 ef	11.63 ± 1.88 cd	11.14 ± 1.02 de	20.35 ± 1.87 cd	86.93 efg
	UV-A	21.93 ± 5.85 c	103.12 ± 5.59 b	22.68 ± 0.38 b	45.79 ± 4.68 a	78.46 ± 9.36 a	271.98 b
	NE	29.97 ± 0.63 ab	81.97 ± 4.75 c	19.09 ± 1.12 b	38.86 ± 4.03 ab	73.11 ± 6.34 a	243.00 bc
	Blue	32.56 ± 4.40 a	46.53 ± 9.26 e	20.18 ± 5.95 b	19.43 ± 3.58 c	26.27 ± 5.30 c	144.97 d
	NE	33.26 ± 4.29 a	64.94 ± 4.39 d	21.48 ± 3.51 b	36.65 ± 0.86 b	60.06 ± 1.44 b	216.39 c
	Green	9.00 ± 1.73 d	28.85 ± 4.70 fg	8.02 ± 1.41 cde	17.64 ± 3.18 cd	30.11 ± 5.31 c	93.62 ef
	NE	9.89 ± 1.80 d	17.97 ± 2.46 gh	6.46 ± 1.34 de	11.98 ± 1.83 de	22.52 ± 3.11 cd	68.82 fg
	Red	23.82 ± 0.74 bc	35.58 ± 2.97 ef	12.40 ± 1.44 c	18.12 ± 1.27 cd	28.42 ± 1.46 c	118.34 de
	NE	19.02 ± 2.33 c	11.10 ± 0.42 h	5.15 ± 0.49 e	8.92 ± 0.34 e	12.68 ± 0.79 d	56.87 g
LSD values		7.93	13.79	5.53	7.19	12.65	36.53

NE indicates not-exposed apple skins. Data indicate averages with standard deviations in triplicate experiments per treatment (*n* = 3). Different alphabets within a column of colorant treatment are significantly differed at *p* < 0.05 on Fisher’s LSD test.

**Table 3:** Accumulation of quercetin glucosides in S2 ‘Fuji’ apple skins after light irradiation with/without MeJA. Unit: mg∙100 g^−1^ DW.

MJ	Light	Quercetin-3-O-Rutinoside	Quercetin-3-O-Galactoside	Quercetin-3-O-Glucoside	Quercetin-3-O-Xyloside	Quercetin-3-O-Rhamnoside	Total Quercetin Derivatives
non-MeJA	UV-B	16.95 ± 0.49 cd	341.16 ± 25.85 a	64.89 ± 2.47 a	112.92 ± 7.76 a	176.42 ± 11.42 a	712.34 a
	NE	20.24 ± 1.03 bc	44.39 ± 4.37 fg	12.82 ± 1.03 fg	27.33 ± 2.00 ef	55.77 ± 5.73 cde	160.55 ef
	UV-A	25.99 ± 0.79 a	266.09 ± 5.25 b	51.74 ± 2.10 b	75.10 ± 1.00 b	100.85 ± 4.06 b	519.77 b
	NE	3.61 ± 1.03 f	52.39 ± 6.37 e	13.52 ± 1.10 f	26.88 ± 2.21 ef	52.03 ± 2.81 cd	148.42 f
	Blue	17.66 ± 0.88 cd	129.69 ± 4.63 d	30.33 ± 1.15 d	36.12 ± 1.49 de	51.94 ± 1.99 de	265.74 d
	NE	24.17 ± 2.90 ab	72.59 ± 8.55 ef	15.36 ± 1.34 f	36.58 ± 2.12 de	69.34 ± 7.27 c	218.04 de
	Green	10.34 ± 1.50 e	213.02 ± 7.50 c	38.99 ± 1.38 c	53.49 ± 1.91 c	59.35 ± 1.93 cd	375.19 c
	NE	14.17 ± 0.41 de	92.71 ± 8.95 e	21.11 ± 1.61 e	42.75 ± 3.25 d	68.47 ± 5.22 cd	239.21 d
	Red	24.11 ± 3.39 ab	102.74 ± 10.71 de	21.54 ± 2.23 e	42.42 ± 4.00 d	70.00 ± 5.58 c	260.81 d
	NE	21.78 ± 1.83 abc	36.09 ± 3.97 g	8.40 ± 1.08 g	22.82 ± 2.29 f	40.32 ± 5.01 e	129.41 f
LSD values		4.97	31.18	4.08	9.89	16.99	62.48
MeJA	UV-B	10.38 ± 0.48 d	248.71 ± 10.66 b	64.18 ± 2.87 a	54.91 ± 2.11 b	72.13 ± 3.47 b	450.31 b
	NE	12.39 ± 0.71 d	28.07 ± 2.07 f	7.41 ± 0.73 f	16.94 ± 1.59 g	33.97 ± 4.68 c	98.78 f
	UV-A	20.10 ± 0.46 bc	152.78 ± 9.65 c	34.52 ± 1.28 c	36.57 ± 1.73 de	39.49 ± 3.65 c	283.46 cd
	NE	10.08 ± 0.45 d	76.50 ± 9.04 e	11.88 ± 1.86 ef	31.55 ± 4.65 ef	46.44 ± 7.20 c	176.45 e
	Blue	19.70 ± 0.71 bc	62.07 ± 2.49 e	16.06 ± 0.52 e	24.79 ± 0.87 fg	32.62 ± 1.31 c	155.24 ef
	NE	21.48 ± 0.64 bc	137.29 ± 3.17 cd	25.87 ± 0.54 d	55.81 ± 1.10 b	78.46 ± 2.60 b	318.91 c
	Green	20.05 ± 1.18 bc	116.48 ± 3.99 d	25.11 ± 0.50 d	48.92 ± 1.86 bc	83.21 ± 1.86 b	293.77 cd
	NE	17.86 ± 1.10 c	75.46 ± 4.98 e	16.86 ± 1.17 e	45.17 ± 2.62 cd	83.63 ± 6.55 b	238.93 d
	Red	21.77 ± 1.60 b	307.97 ± 24.81 a	42.29 ± 3.66 b	100.69 ± 7.38 a	142.37 ± 11.65 a	615.09 a
	NE	33.06 ± 3.31 a	32.44 ± 3.34 f	9.45 ± 1.63 f	23.56 ± 1.63 fg	46.48 ± 2.29 c	144.99 ef
LSD values		3.87	28.99	5.27	9.26	15.96	59.92

NE indicates not-exposed apple skins. Data indicate averages with standard deviations in triplicate experiments per treatment (*n* = 3). Different alphabets within a column of colorant treatment are significantly differed at *p* < 0.05 on Fisher’s LSD test.

## Data Availability

Not applicable.

## Supplementary Materials

The following supporting information can be downloaded at: https://www.mdpi.com/article/10.3390/ijms23031722/s1.
