# Supplementary material for: Pigmentation and Flavonoid Metabolite Diversity in Immature ‘Fuji’ Apple Fruits in Response to Lights and Methyl Jasmonate
Source: Int J Mol Sci. 2022 Feb 2;23(3):1722. doi: 10.3390/ijms23031722 (PMC8836130; doi:10.3390/ijms23031722)
Supplement: Supplementary file 1 [file ijms-23-01722-s001.zip › ijms-1552334-supplementary.pdf]

Supplementary Materials

Table S1. The information of primer sequences used in metabolic gene expressions of anthocyanin biosynthesis.

Name	Direction	Sequence
MdCHS	F [z]	GGA GAC AAC TGG AGA AGG ACT GGA A
	R [y]	CGA CAT TGA TAC TGG TGT CTT CA
MdCHI	F	GGG ATA ACC TCG CGG CCA AA
	R	GCA TCC ATG CCG GAA GCT ACA A
MdF3H	F	TGG AAG CTT GTG AGG ACT GGG GT
	R	CTC CTC CGA TGG CAA ATC AAA GA
MdDFR2	F	GAT AGG GTT TGA GTT CAA GTA
	R	CGG CCG GAA TCG GAA TCA ATC
MdLDOX	F	CCA AGT GAA GCG GGT TGT GCT
	R	CAA AGC AGG CGG ACA GGA GTA GC
MdFLS	F	ACG AGC AAC CGG GAA TCA CAA CTG
	R	CCC AGT TGG AGC TGG CCT CAG TA
MdUFGT	F	CCA CCG CCC TTC CAA ACA CTC T
	R	CAC CCT TAT GTT ACG CGG CAT GT
MdMYB10	F	TGC CTG GAC TCG AGA GGA AGA CA
	R	CCT GTT TCC CAA AAG CCT GTG AA
MdACS6	F	AGT GGA TTA ATT CGC TCT TAT GGT G
	R	GAA CAT CCT CTG GAG TCA AAG TAG
MdActin	F	TGA CCG AAT GAG CAA GGA AAT TAC T
	R	TAC TCA GCT TTG GCA ATC CAC ATC

[z] and [y] indicate forward (5′ → 3′) and reverse (3′ → 5′), respectively.

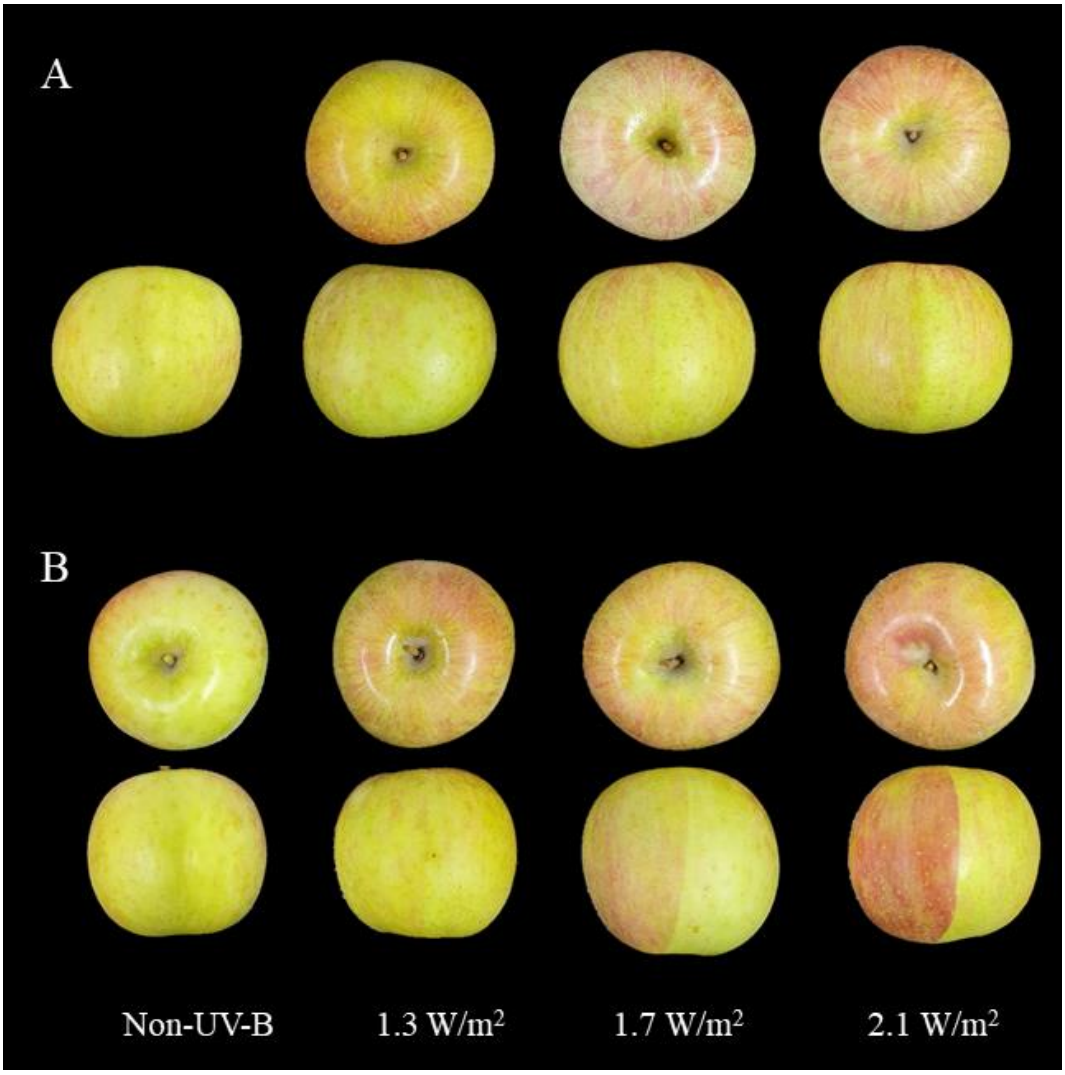

Figure S1. Pigmentation of S2 'Fuji' apples by the effects of UV-B intensities either without MeJA (**A**) or with MeJA (**B**) after 3 days of UV-B irradiation. The status of S2 apples were 145 days after full blooming and just showed the initiation of apple pigmentation on the apple trees.
